# Performance behaviour of agro-waste based gypsum hollow blocks for partition walls

**DOI:** 10.1038/s41598-022-07057-y

**Published:** 2022-02-25

**Authors:** Siddharth Singh, Soumitra Maiti, Ravindra Singh Bisht, Nagesh Babu Balam, Rakesh Solanki, Ajay Chourasia, Soraj Kumar Panigrahi

**Affiliations:** 1 Acoustics Instrumentation and Mechanical Systems Group, CSIR-Central Building Research Institute, Roorkee, 247667 India; 2 Environmental Science and Technology Group, CSIR-Central Building Research Institute, Roorkee, 247667 India; 3 Efficiency of Buildings Group, CSIR-Central Building Research Institute, Roorkee, 247667 India; 4 Fire Division Group, CSIR-Central Building Research Institute, Roorkee, 247667 India; 5 Structural Engineering Group, CSIR-Central Building Research Institute, Roorkee, 247667 India

**Keywords:** Engineering, Structural materials, Mechanical properties

## Abstract

Crop residue management is a massive problem in the agriculture sector. Agricultural waste in the form of stubble which is usually burnt in the farm fields, causes severe air pollution and poses a threat to the environment. The present study investigates the addition of agro-waste (rice straw) in gypsum hollow-core blocks for partition walls. Various compositions of agro-waste-based gypsum samples have been studied for compressive strength, thermal, sound absorption, sound transmission loss, and fire-resistant properties. The addition of rice straw in gypsum reduces the density and compressive strength of the test sample, thus making it lightweight for non-load bearing wall application. The thermal conductivity of the rice straw added gypsum samples show a decrease in thermal conductivity from 0.2 to 0.11 W/m K. Acoustic properties viz., noise reduction coefficient (NRC) increases from 25 to 45% with increase in rice straw addition and a decreasing trend in sound transmission class (STC) from 37 to 28 dB. The fire-resistant properties viz., surface spread of flame, and fire propagation index test have shown good fire-resistant properties. The agro-waste-based hollow gypsum blocks may be used as a promising material for drywall partitions owing to its thermal insulation, low density, good acoustic and fire-resistant properties.

## Introduction

Crop residue management is a serious challenge in India and worldwide as farmers struggle to manage economic sustainability, to adopt clean agriculture practices and meet long-term production requirements. One of the approaches for increasing revenue and production is to harvest and use crop residues as cattle feed or feedstock for biofuel production. These agro-wastes are also required in mitigating soil erosion, recycling of essential plant nutrients for future crops, and providing soil organic matter^1^.

In India, approximately 500 million tons of crop residue are generated annually^2^. The majority of crop residue is used as fodder, fuel for domestic and industrial purposes for energy consumption^2, 3^. The majority of residues from agricultural commodities viz., rice, wheat, maize, and pulses are used as cattle feed, while cotton, chili, pulses, and oilseeds are used as fuel for household requirement. Rice husk is primarily used as fuel in boilers and whereas, bagasse is mainly utilised in energy or paper industries^3–5^.

The amount of surplus agricultural residue generated in India is about 220 million tons per year. Most of which accounts for rice and wheat residue, out of which around 60% is burnt on field^1, 4^. The burning of these crop residues causes severe air pollution in the vicinity and as well as far areas of the region. According to literature, burning one ton of straw results in the release of particulate matters (PM_2.5_): 3 kg, carbon monoxide: 60 kg, carbon dioxide: approximately 1000 kg, ash: 200 kg, and sulphur oxide: 2 kg^5^.

Extensive research has been done on the utilisation of agricultural waste as a substitute in building materials. Many agrarian waste materials are already used in concrete as replacement alternatives for cement, fine aggregate, coarse aggregate, and reinforcing materials^6,7^.

Other residues obtained from sugarcane bagasse ash, groundnut shell, oyster shell, sawdust, giant reed ash, rice husk ash, cork and tobacco wastes are used as fine aggregates in concrete. These are used as partial replacement of fine aggregate, which provides additional pozzolanic property in concrete^6, 7^.

Agro-waste in the form of straw residues has also been a subject of intensive study by many researchers in recent years. Evaluation of various percentages and sizes of straw fibre incorporation on the engineering properties of unfired earth bricks has been done extensively and studied by various researchers. The literature reveals that the increase of straw percentage results in reduced compressive strength, density, and thermal conductivity of unfired earth bricks^8–13^. Few researchers have also studied the incorporation of agro-waste viz., coconut shell, oil palm shell as an alternative to coarse natural aggregates^14^. A study on the combined properties of clay bricks with rice husk has shown promising results which can be potentially used in the production of lightweight bricks^15^. The use of coconut, bagasse, and palm oil fibres in soil blocks has also been studied and mentioned elsewhere^16^. Marques et al.^17^ reported the vibrational and acoustic behaviour of polymer-based composite materials produced with rice husk and recycled rubber granules. The composite boards show improved performance for impact sound insulation. Results also validate that the composites produced from rice husk and recycled rubber granules mitigate vibration in buildings. A similar study by the same author has been done on rice husk and cork granules-based composites. The results show the thermal conductivity of the composites vary over the range of 0.0495 to 0.0798 W/m K and maximum sound absorption coefficient 95% at 1250 Hz^17^.

Another study on rice husk panels for building applications has been done by Buratti et al.^18^ regarding thermal, acoustic and environmental characterisation of recycled wastes. The waste includes paper residues, rubber granules, cork scraps, wood scraps, and coffee chaff, etc. The thermal conductivity of 0.07 W/m K and sound absorption coefficient of 0.87 at 2500 Hz was noted for rice husk panels, and the values are comparable with other recycled products described elsewhere^18, 19^. Many researchers have studied the application of agro-waste ashes from various resources viz., wood, rice husk, rice/wheat straw ash, sugarcane bagasse ash, etc., as a replacement for cement and aggregates^14, 20–22^. Biomass obtained from agricultural residues has potential application in the production of biofuels, electricity, and heat, which can be utilised in various domestic and industrial applications^23^.

Very few literature is available on gypsum-based agro-wastes hollow building products. In a study by Junior et al. reported the acoustic properties of hollow gypsum panels as wall partitions. But they have not reported studies regarding compressive strength, fire resistance, water absorption, and thermal conductivity of hollow gypsum panels^24^. A similar kind of study on sound transmission loss on clay hollow brick wall has been done by Granzotto and co-workers^25^. According to the literature, dimension, geometry, void structure, and the fraction of hollow bricks were considered in the experimental and analytical methods of sound transmission loss study. In a review study by Jia et al. on fibre reinforced gypsum composites in building construction, various aspects of gypsum sources and their properties utilised in construction have been mentioned in detail^26^. Some of the drawbacks of gypsum composites include brittle and hygroscopic nature, low thermal and sound insulation, and shrinkage during fire exposure usually restricts its application. Most of these drawbacks may be improved by the introduction of either synthetic or natural fibres in gypsum products, thus making them more versatile in building applications. Examples of synthetic fibres include E-glass fibres, alkali-resistant glass fibres, polypropylene fibres. Natural fibre sources include coir, jute, hemp, sisal, and rice can be used to enhance the properties of gypsum products^26^.

The present study aims to investigate the effect of the incorporation of rice straw in gypsum hollow panels/blocks for non-load bearing wall application. The investigated behaviours include mechanical strength, sound absorption, sound transmission loss, surface spread of flame and fire propagation index, and thermal conductivity. The study of crop residue in the form of rice straw utilisation in gypsum plaster boards has been done by various researchers but with a limited scope covering only a few of the investigations as mentioned. Here, an in-depth study of mentioned properties has been done on straw gypsum hollow panels that provide adequate mechanical strength, show good thermal and sound insulation along with fire-resistant properties. Hollow cores will help in reducing the overall weight of the blocks, which may be filled with highly lightweight thermal insulating materials viz., glass or mineral wool to provide additional thermal insulation. These cores may also be utilised in electrical conduits. Agro-waste gypsum blocks have the potential application in building materials, which will help in reducing air pollution that arises from burning agro-wastes in the farm fields.

## Experimental procedure

The experimental program involved casting and characterising the material properties and mix-design preparation for agro-waste-added hollow gypsum blocks for evaluation of various parameters.

### Materials and mixture proportion

**Figure 1:**
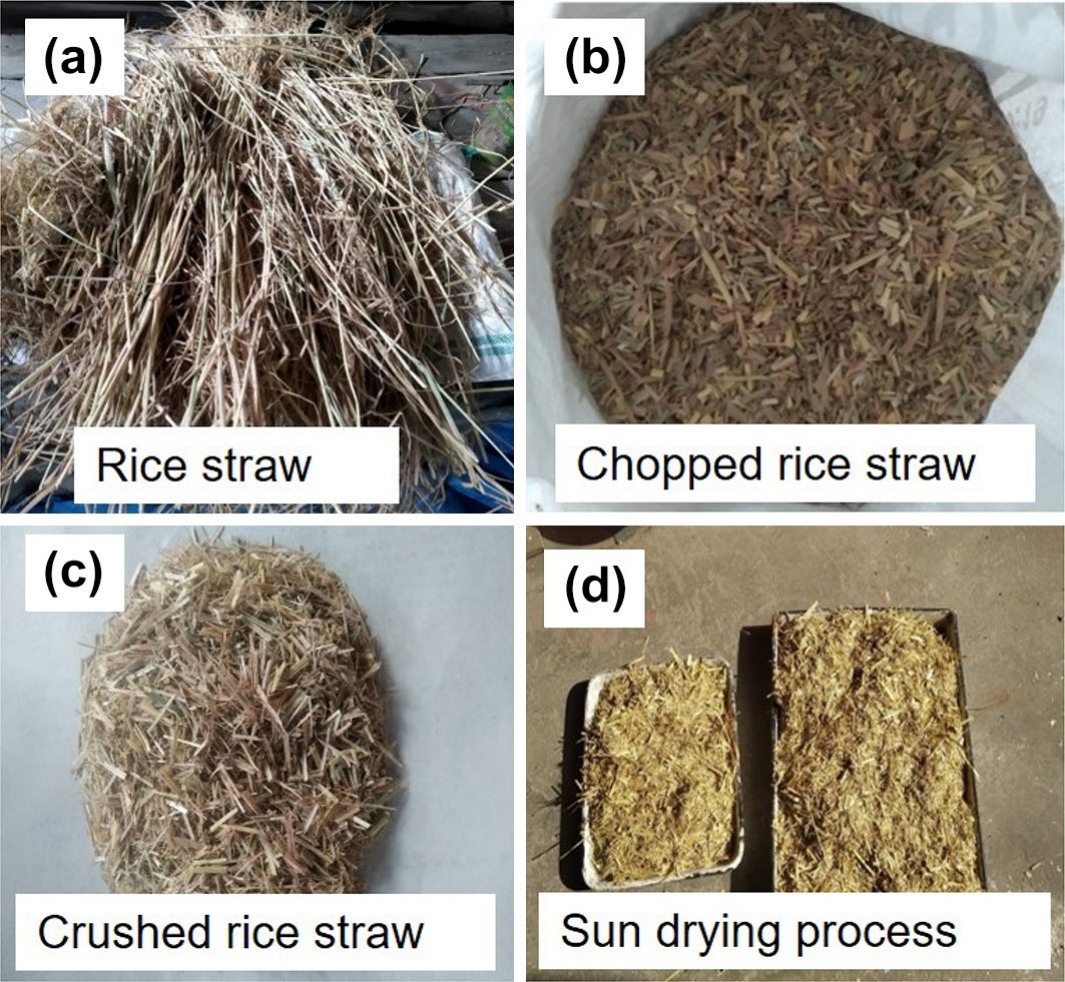
(**a**) Collected rice straws from farm fields; (**b**), (**c**) chopped and crushed rice straw; and (**d**) sun drying process for moisture removal.

For the present study, the agricultural waste as rice straw from local farm fields was collected and used as raw material. The straws were approximately 1 m in length shown in Fig. 1a when collected from the field. The straws were dried under sunlight (35–40 °C) for 7 days to remove moisture content. After the drying process, the straw bundles were chopped into small pieces of 1 inch or less depending upon the requirement. The chopped straws were further dried under sunlight for 3 days to further remove moisture content. Figure 1b,c show the chopped and crushed rice straws of 5 to 10 mm length and 1 to 3 mm thickness, and sun-drying process in Fig. 1d. The effect of straw size on physical properties of casted straw gypsum samples have been discussed in-depth in subsequent sections. Table 1 shows the physical properties of chopped rice straw and hemihydrate gypsum.

**Table 1:** Physical properties of rice straw and gypsum.

Physical properties	Gypsum	Agro-waste (rice straw)
Colour	White	Brown/yellow
Weight	15–20 kg; 4 hollow core	–
	4–6 kg; 2 hollow core	
Thermal conductivity (W/m K)	0.2–0.24	0.07 to 0.09
Loose density (kg/m^3^)	800–1000	90–110
Compact density (kg/m^3^)	1200–1400	–
Fire resistance	Non-combustible	Combustible
Organic/volatile matter	Maximum 0.1%	Organic**/**60–70%
Biological	Rot proof	Highly susceptible
Moisture content	10.5%	11%
Chopped straw size (length and thickness)	–	25 mm and 3–5 mm
Crushed straw size	–	5–10 mm and 1–3 mm

#### Gypsum agro-waste mix design

**Figure 2:**
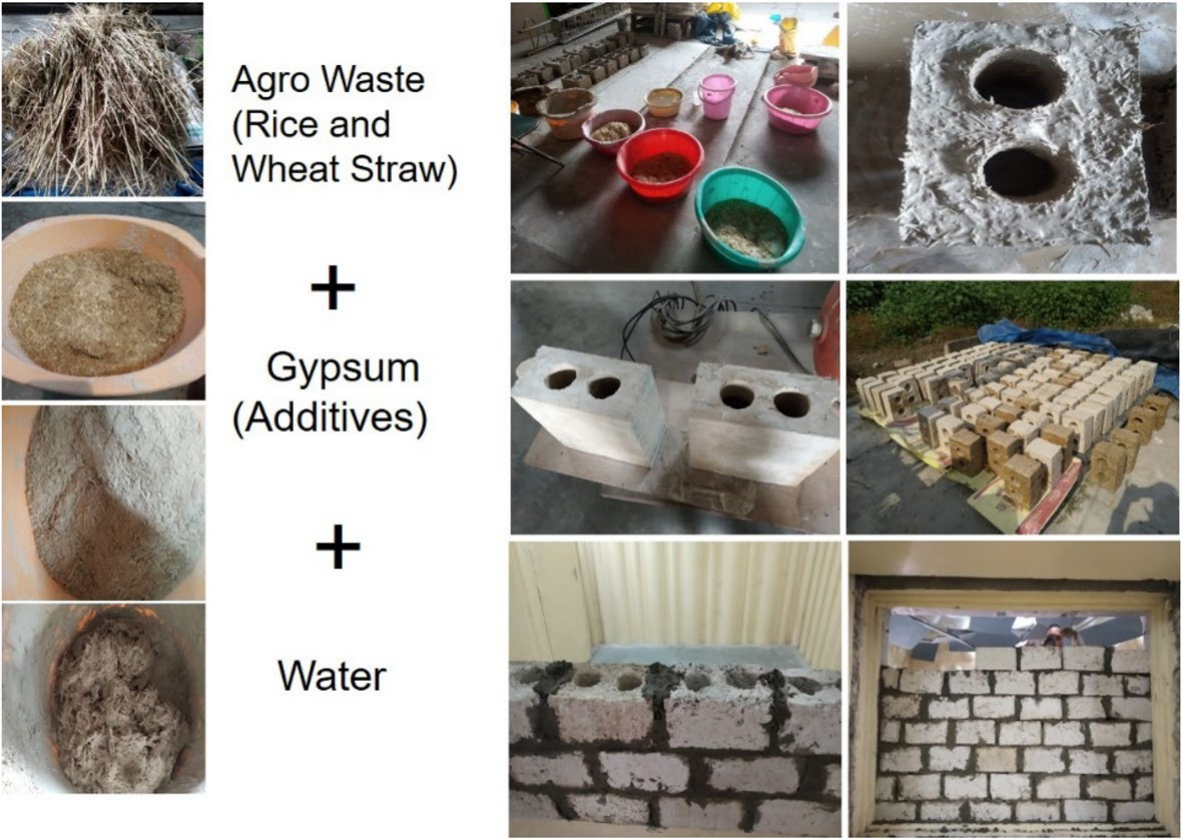
Process, casting, sun drying and wall construction of agro-waste gypsum blocks.

**Figure 3:**
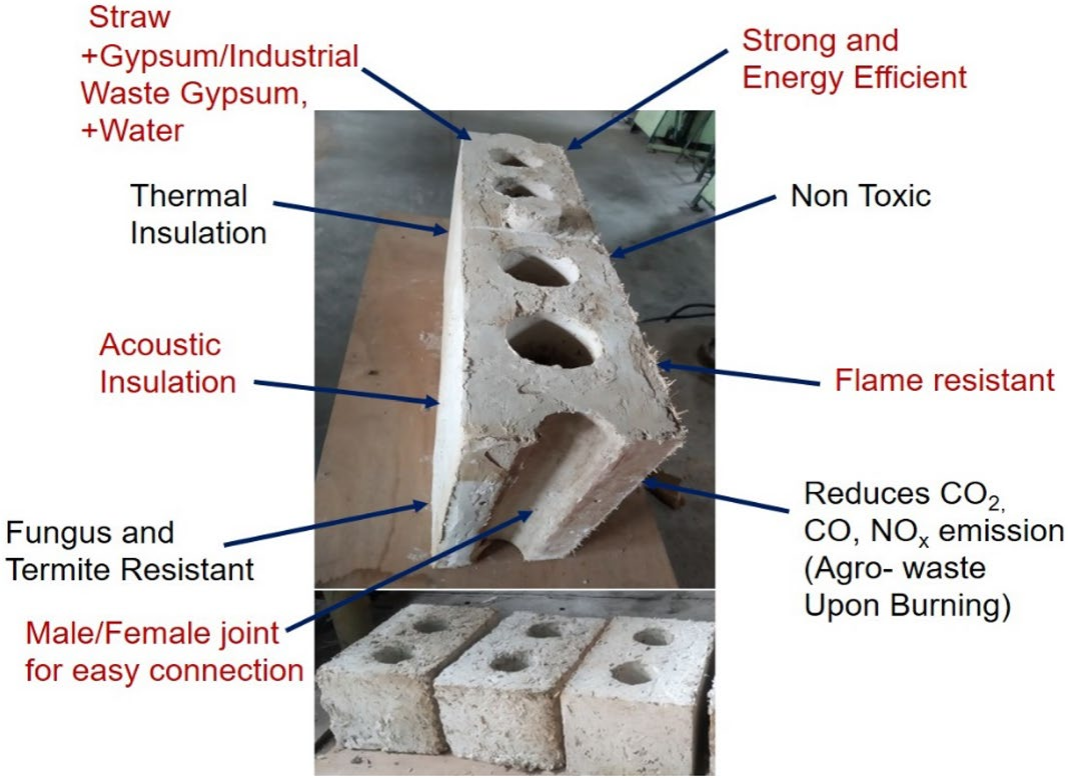
Features of agro-waste gypsum panel/blocks.

For the present study, rice straw as agro-waste has been incorporated in gypsum (with retarders as additives) as 5, 10, 15, 20, and 25 wt% with water to gypsum ratio being kept at 0.6. Chopped rice straw in gypsum was mixed and casted samples were characterised for various experiments. A four-core hollow panel of size 600 mm × 300 mm × 150 mm with 5 wt% addition of rice straw with a hollow core diameter of 70 mm was casted for reference purpose. Typical male–female joint for joining of blocks was also provided for ease of panel jointing. The total dry weight of the panel weighs around 25 kg. Two-core hollow blocks with 300 mm × 200 mm × 150 mm were casted for prototype wall construction and sound transmission loss study. The block dimensions were in accordance with Indian standard (IS) code^27^. Figure 2 shows a representation of the casting process of hollow blocks and Fig. 3 shows the salient features of proposed hollow blocks/panel.

### Specimens, test methods, and test setup

#### Compressive strength, density, porosity and water absorption tests

In the present study, various characterisation were done to evaluate the properties of agro-waste gypsum samples. The mechanical performance of the gypsum samples was analysed by compressive strength test, dry density test, water absorption test, nail retention, and free moisture test.

Compressive strength and dry density measurements were performed in accordance with IS code^28, 29^. Cubes of 50 mm × 50 mm × 50 mm were casted and retained in the moist air for of 24 h. The cubes were then kept in an oven maintained at a temperature of 45 °C with relative humidity below 50%. Dried cubes were weighed once each day up to 7 days. The compressive strength was done on five samples, and the average was reported as the final compressive strength.

Water absorption and moisture content test were done according to IS code^28, 29^. From each board/panel, a specimen of 175 mm × 75 mm was taken. The sample was weighed to within 1 gm and then stored at a temperature of 27 ± 2 °C in an atmosphere having a relative humidity (R.H.) of 65 ± 2%. The specimen was weighed until the mass had become constant to within 0.1% of the initial weight. After this, the sample was completely immersed in water at 27 ± 2 °C for 24 h, with at least 30 mm height of water over the top of the specimen. The sample was taken out and weighed (W_1_) after removing additional moisture with a damp cloth. Then the specimen was placed in an air oven to 105 °C and retained at that temperature. The samples were dried until the mass of the sample is constant (within ± 0.1% of the mass of the sample) and this mass (W_2_) was recorded for each sample. The percentage absorption was calculated as follows according to Eq. (1)^28^:

1 $$\text{Percentage} \; \text{absorption}= \frac{\left[{W}_{1}- {W}_{2} \right]}{\left[{W}_{2}\right]}$$

where ${W}_{1}$ is mass of specimen after absorption, and ${W}_{2}$ is mass of sample after heating.

For moisture content, the two-core hollow block of 300 mm × 200 mm × 150 mm with 5 wt% agro-waste was tested. The test specimen was weighed, and the mass (M_1_) was recorded. The specimen was dried to constant mass in an air-oven raised and maintained to 105 °C temperature. The specimen was weighed at intervals of 24-h until constant mass (within ± 0–1% of the mass of the specimen) was attained, and this mass (M_2_) was recorded for each specimen. The percentage moisture content was calculated from the following formula for each specimen as per Eq. (2)^28^:

2 $$\text{Percentage} \; \text{moisture} \; \text{content}= \frac{\left[{M}_{1}- {M}_{2} \right]}{\left[{M}_{2}\right]}$$

where, ${M}_{1}$ is initial mass of specimen, and ${M}_{2}$ is dry mass of specimen after heating.

Nail retention test was done on gypsum agro-waste blocks prototype wall according to IS code^28^. Copper roofing or galvanised iron nail of 3 mm diameter was driven to a depth of 25 mm in the block. The nail was then pulled out axially and the force required for pull-out was measured in newton ($N$).

### Thermal, acoustic and fire characterisation

Solid agro-wastes panels of size 300 mm × 300 mm × 25 mm with various agro-waste content for thermal conductivity $k$ was measured by guarded hot plate apparatus in accordance with IS code^30^. The thermal conductivity was measured at a mean temperature of 40 °C. Agro-waste gypsum panels were casted and dried at 45 °C for thermal conductivity testing.

The sound absorption and sound transmission loss (STL) measurement of gypsum and agro-waste gypsum samples were tested by impedance tube method according to ASTM code^31^.

The noise reduction coefficient (NRC) and sound absorption average (SAA) for agro-waste gypsum samples were measured and analysed. Both the values are single number ratings exhibiting the level of sound absorption by a particular sample. The NRC value is the average of the sound absorption coefficients at 1/3rd octave frequencies of 250, 500, 1000 and 2000 Hz. Whereas, the SAA value is the average of the sound absorption coefficients at twelve 1/3rd octave frequencies ranging from 200 to 2500 Hz. The normal incidence sound absorption coefficient, ${\alpha }_{n}$, is given by Eq. (3)^31^:

3 $${\alpha }_{n}=1- {\left|\Gamma \right|}^{2}$$

where, $\Gamma$ is reflection coefficient and ${\alpha }_{n}$ is normal incidence sound absorption coefficient.

The STL and corresponding sound transmission class (STC) values of agro-waste gypsum samples were determined by impedance tube according to ASTM standard^32, 33^. The STL tests were also carried out by reverberation method, consist of two reverberation chambers, of volumes 120 m^3^ and 30 m^3^, respectively, isolated by a fixed concrete frame. The walls and slabs of the chamber are 40 cm thick and were designed in such a way that no wall is parallel to each other. The floors and walls of the reverberating rooms were coated with reflective paint to provide maximum reflection. Each chamber has access with 40 mm-thick, wooden steel plated double doors. STL tests of agro waste gypsum were conducted with the following equipment: sound level meter (SLM) SC 420 (CESVA); omnidirectional sound source: model BP 012 (CESVA); noise generator with power amplifier: model AP 602 (CESVA). The STL data were collected at 1/3rd octave band frequencies ranging from 100 to 6300 Hz.

**Figure 4:**
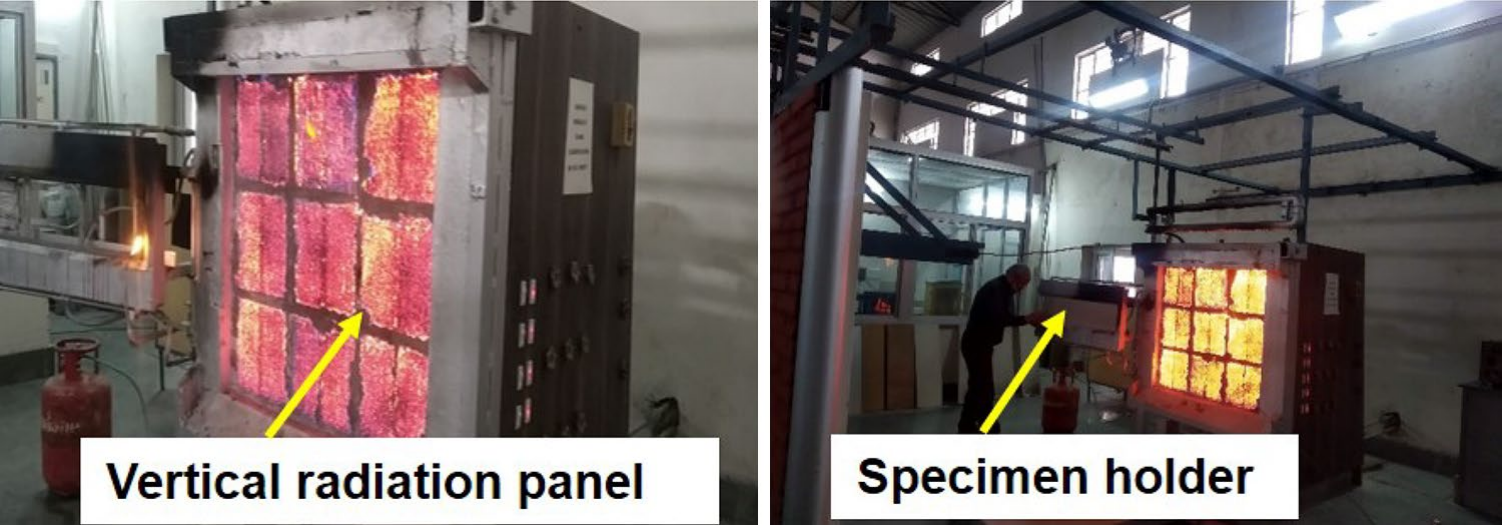
Surface spread of flame of products apparatus in accordance with BS 476 part 7.

Surface spread of flame test was carried out on 5 wt% of agro-waste gypsum samples (03 nos.) of specimen size of 900 mm × 270 mm × 30 mm in accordance with British standard (BS)^34^ as shown in Fig. 4. The test method specifies measuring the lateral spread of flame along the surface of a test specimen. It gives an in-depth analysis for comparing the performances of flat materials, or assemblies being used as interior lining materials used in building. The test specimen was placed in a vertical test position adjacent to the radiation panel within 5 s of igniting a pilot flame. The test specimen was set on fire by a pilot flame, and this flame was extinguished after one minute. Air and gas flow is adjusted so that there is uniform radiant exist over the face of the test specimen. The behaviour of the specimen was carefully observed throughout the evaluation for flashing, transitory flaming, debris falling, flaming, non-flaming capabilities or any other deformation.

The fire propagation index (FPI) test, provides a comparative measure of the contribution to the growth of fire tested for agro-waste gypsum samples. It is primarily intended for the assessment of the performance of internal wall and ceiling linings. According to BS code^35^, this method specifies a test for providing a comparative measure of a flat test specimen's contribution to the growth of a fire. It considers the combined effect of factors such as ignition characteristics, amount and rate of heat release, thermal properties of the product concerning its ability to accelerate the rate of fire growth.

For fire propagation index evaluation, a calibration run was conducted by exposing non-combustible board of specified properties. Similar runs were repeated for the specimens of materials keeping the gas flow rate to the flame burner and power supply to the radiant heaters, same as in the case of the calibration run. The time–temperature values for the materials and those obtained during the calibration were recorded. The time–temperature values were determined using the calibration board for 20 min. The temperatures were also recorded with time for the specimens of the materials in the same way as for the calibration board for 20 min. Three samples of size 223 mm × 222 mm × 29 mm were tested for fire propagation. The fire propagation index ($I$) may be defined according to Eq. (4)^35^: 

4 $${I=i}_{1}+{i}_{2}+{i}_{3}$$

where, ${i}_{1}$, ${i}_{2}$, ${i}_{3}$ are the sub-indices.

### Prototype wall construction

Test wall of 1.8 m × 1.2 m using two-core hollow blocks was constructed in reverberation chamber, and a prototype wall of 2.6 m × 2.2 m was built for STL, STC, thermal admittance, nail retention, and performance evaluation testing. The blocks were joined by cement mortar with cement to sand ratio of 1:6. The blocks were joined carefully such that there were no openings between them.

## Results and discussion

### Effect of rice straw on mechanical properties

**Figure 5:**
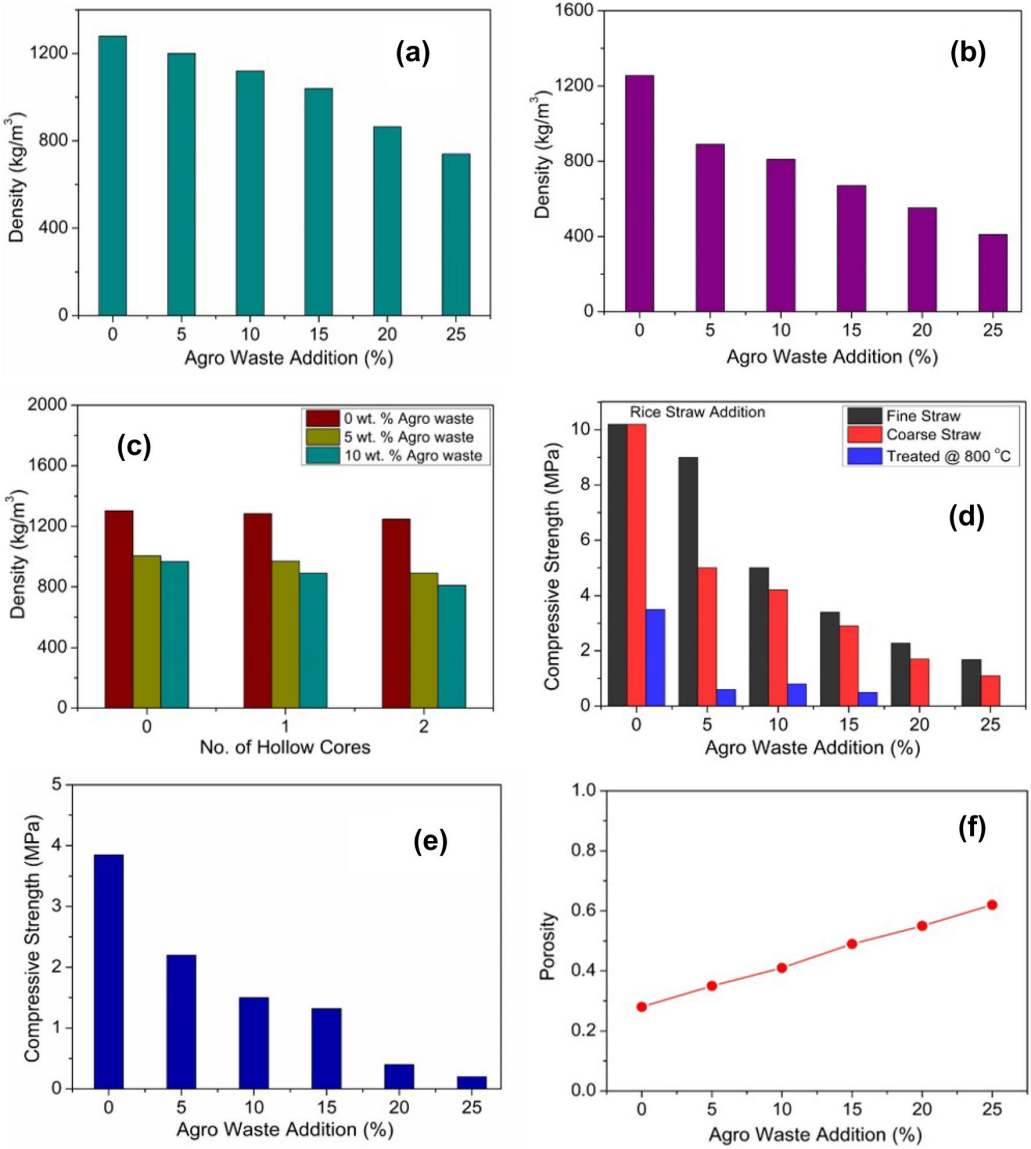
(**a**) Density variation of rice straw added gypsum cubes; (**b**) density variation of two-core hollow agro waste gypsum blocks; (**c**) density plot of rice straw based gypsum blocks with hollow-core variation; (**d**) compressive strength of rice straw added gypsum cubes of various straw size and treatment at 800 °C; (**e**) compressive strength of rice straw added gypsum two-core hollow blocks; (**f**) porosity variation of gypsum and rice straw based samples.

Figure 5a shows the compact dry density variation of 0 to 25 wt% addition of rice straw in gypsum cubes (50 × 50 × 50 mm^3^). As evident from the bar chart, the density of the cubes reduces with the increase in agro-waste addition from 1270 to 830 kg/m^3^. This is attributed to the low density of rice straw of 90–110 kg/m^3^. Figure 5b shows the dry density of two-core hollow gypsum blocks with varying agro-waste compositions. From the plot, it is evident that with increasing agro-waste content the dry density of hollow core gypsum blocks decreases up to 450 kg/m^3^. Whereas, Fig. 5c shows density variation of 0, 5 and 10 wt% rice straw addition with 0, 1, and 2 number of hollow cores. As observed from Fig. 5c, the density of blocks reduces as the number of cores and percentage of agro-waste increases. The effect of fine and coarse straw size plays a vital role in compressive strength determination, as shown in Fig. 5d. The coarse straw addition leads to lower compressive strength as compared to fine straw. The compressive strength of gypsum cubes for coarse straw addition varies over the range of 5 to 1 MPa, whereas, for fine straw agro-wastes, the compressive strength varies from 10 to 2 MPa. Rice straw-based gypsum samples treated at 800 °C for 1-h shows the compressive strength of 3 MPa and reduces to 0.5 MPa for higher rice-straw percent (15 wt%). Compressive strength of various weight percent of rice straw added two-core gypsum hollow blocks is shown in Fig. 5e. From Fig. 5e it can be observed that with the increase in rice straw, the compressive strength of the hollow blocks reduces with maximum strength of 3.8 MPa for 0 wt% agro-waste addition and 0.5 MPa for 25 wt% straw addition. For 5 wt% addition of straw, the compressive strength is 2.2 MPa which has been used in prototype wall construction. Figure 5f shows the porosity of different gypsum agro-waste compositions. The porosity is around 30% for pure gypsum samples and increases up to 65% as the rice straw content increases. The effect of porosity may also be seen and validated for NRC and compressive strength of the straw-gypsum samples. The 24-h water absorption test of 5 wt% rice straw added gypsum sample has been done in accordance with IS code^28^, and the value has been found to be 22% calculated according to Eq. (1). The maximum prescribed value for water absorption is 15% for gypsum samples^26, 27^. Due to the presence of rice straw, the water absorption is high as compared to the prescribed limit. After water repellent coating on the hollow gypsum blocks, the water absorption capacity was reduced over a range of 15–18%. Compared with other building materials viz., fired clay bricks, hollow clay bricks, these materials have higher compressive strength of 15 MPa and 3.5 MPa, respectively, compared to hollow gypsum blocks utilised in the present study^36, 37^. According to the IS code for non-load bearing wall applications minimum of 0.5 MPa strength is required^27^. Therefore, the hollow blocks used in the present study with 5 wt% straw addition can be utilised in non-load bearing wall applications. A prototype wall constructed from 5 wt% rice straw and gypsum has been demonstrated in the current study and mentioned in detail in “Prototype wall demonstration” section. Though the compressive strength of straw added gypsum hollow blocks is less than the pure gypsum and solid blocks, but high thermal and sound insulation properties along with fire resistance behaviour have been achieved by incorporating agro-waste (straw) in the gypsum matrix.

### Thermal conductivity of agro-waste gypsum samples

**Figure 6:**
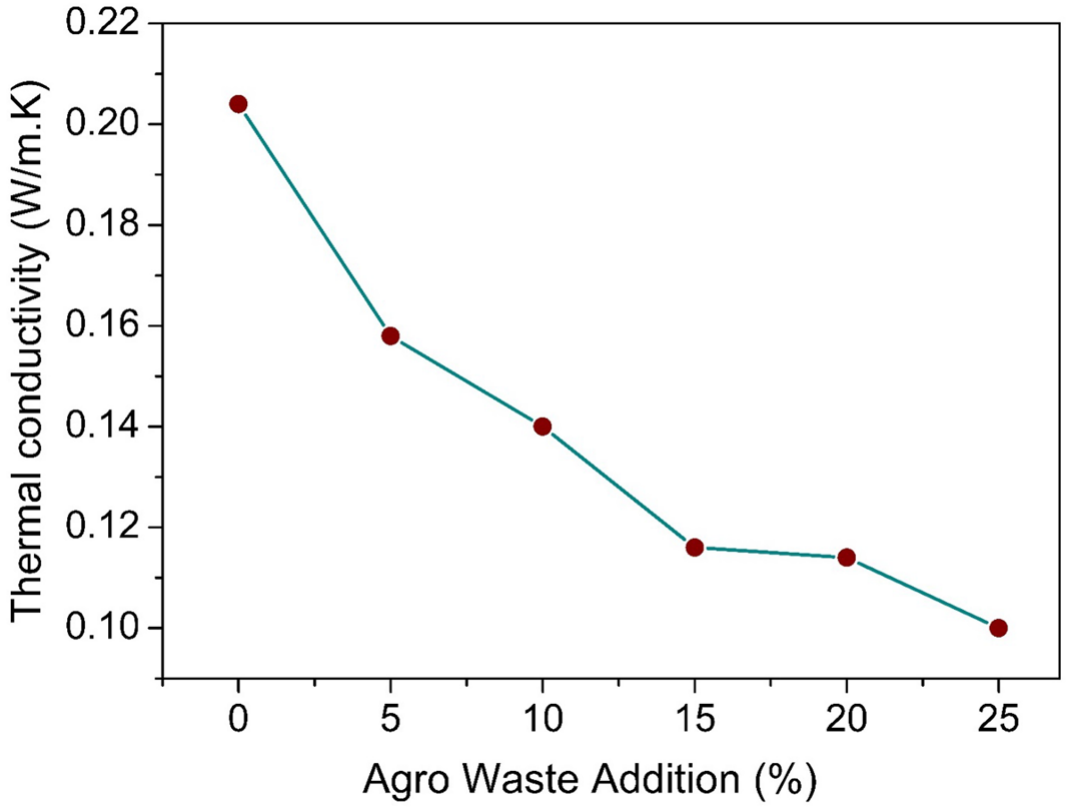
Thermal conductivity variation of rice straw added gypsum samples.

Along with the mechanical performance of agro-waste gypsum samples, thermal property also play an equally important role. Figure 6 shows the thermal conductivity variation of gypsum samples with rice straw addition. As evident from Fig. 6, upon increasing the rice straw content in the gypsum sample, the thermal conductivity decreases. For pure gypsum sample, the thermal conductivity is 0.2 W/m K, and 0.11 W/m K for 25 wt% rice straw added sample. The thermal conductivity of rice straw is around ~ 0.09 W/m K for 10% moisture content. According to a study by Chindaprasirt et al.^38^, the thermal conductivity of the gypsum sample has been reported with a thermal conductivity of 0.35–0.4 W/m K. With organic fibre addition in the gypsum matrix up to 0.5 wt%, the thermal conductivity decreases to 0.26 W/m K^38^. The decrement in the thermal conductivity could be related with increased porosity of the samples and the low thermal conductivity of the fibres. The present study also validates that the addition of rice straw enhances the thermal insulation of the material. Though the thermal conductivity of the rice straw incorporated gypsum is higher than rice straw, but it is significantly lower than burnt clay bricks and concrete walls having $k$ value of 0.8 and 1.26 W/m K, respectively, which are still used as major building materials in construction^39^.

### Estimation of noise reduction coefficient and sound transmission class

**Figure 7:**
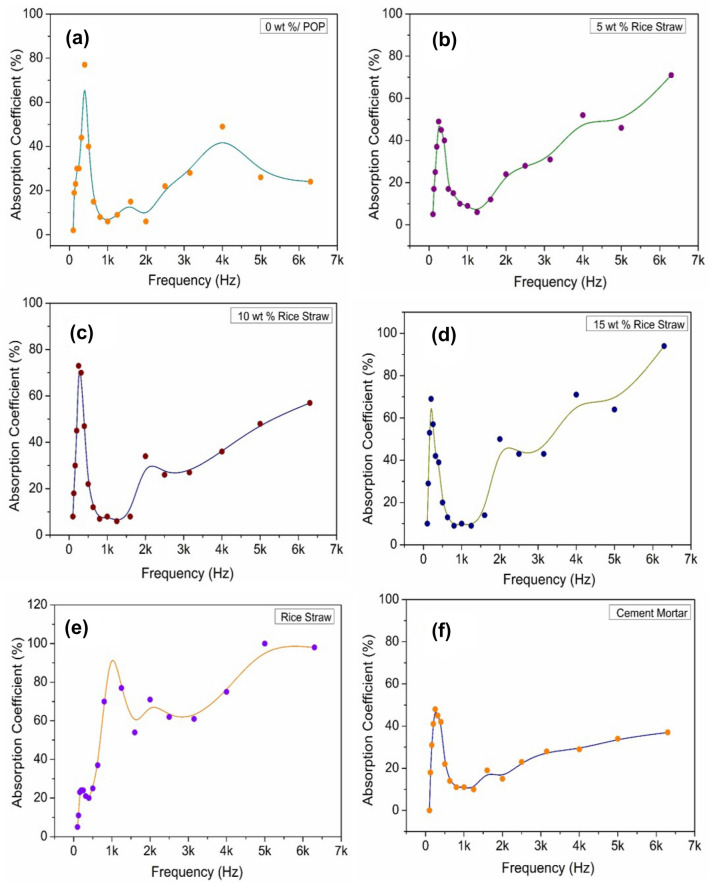
(**a**–**d**) Sound absorption coefficients of various agro-waste based gypsum samples; (**e**) rice straw; (**f**) cement mortar.

**Figure 8:**
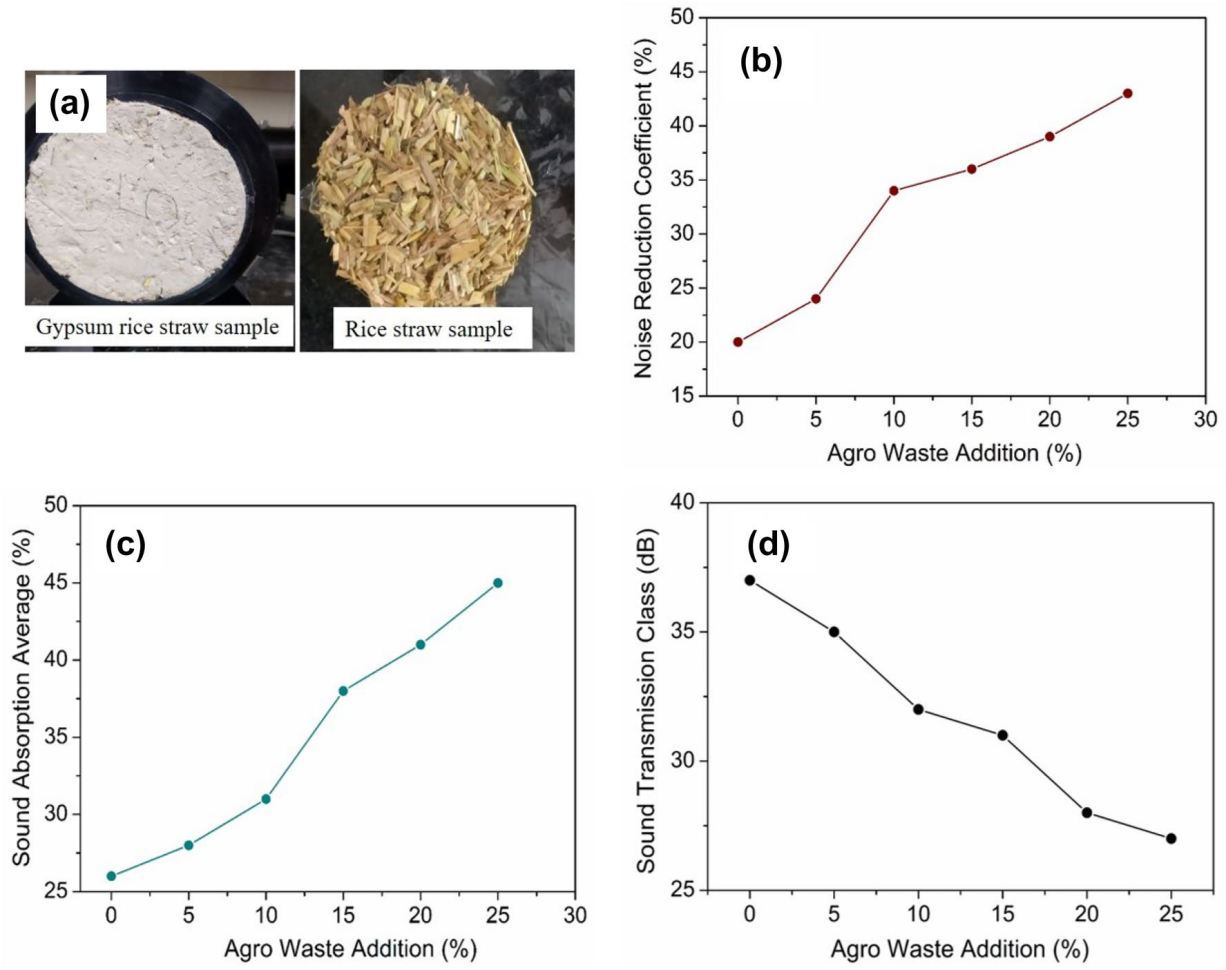
(**a**) Agro-waste gypsum samples for sound absorption and STL measurement; (**b**) (**c**) Noise reduction and Sound absorption average coefficients of various agro-waste gypsum samples; (**d**) Sound transmission class of various agro-waste gypsum samples.

Any material in a wall partition must have good sound insulation features to provide good sound insulation to its occupants. To analyse the acoustic features viz., sound absorption and STL of agro-waste gypsum samples, NRC and STL were measured over the frequency range of 100–6300 Hz using an acoustic impedance tube (HOLMARC). The measurement results are shown in Fig. 7. For comparison purposes, sound absorption characteristics of rice straw and cement mortar are also studied. From Fig. 7a–d, it is evident that the absorption coefficient of 0 to 15 wt% straw added gypsum samples become highest in the frequency range of 150–400 Hz. At this frequency range, the absorption coefficient is found to be over the range of 50–80%. Whereas, for rice straw and cement mortar samples in Fig. 7e,f the highest NRC is 90% and 50% respectively. Figure 8a shows the 20 mm thick and 100 mm diameter straw added gypsum samples for NRC and STC testing. Figure 8b,c show the NRC and SAA of gypsum agro-waste samples. As evident from Fig. 8b,c, the NRC and SAA values increases from 25 to 45% upon addition of rice straw. This increment is attributed to the enhancement in porosity of the sample with increasing straw content.

**Figure 9:**
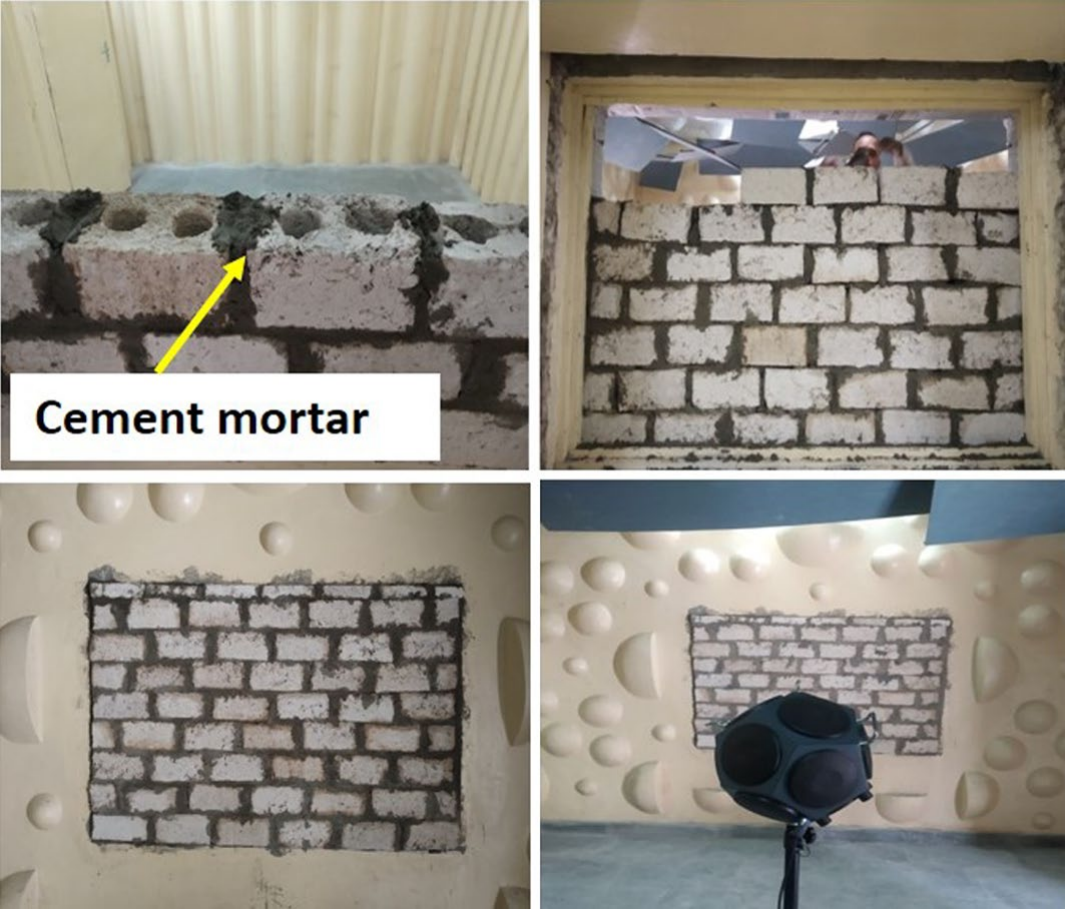
Test wall construction for sound transmission loss in the reverberation chamber.

**Figure 10:**
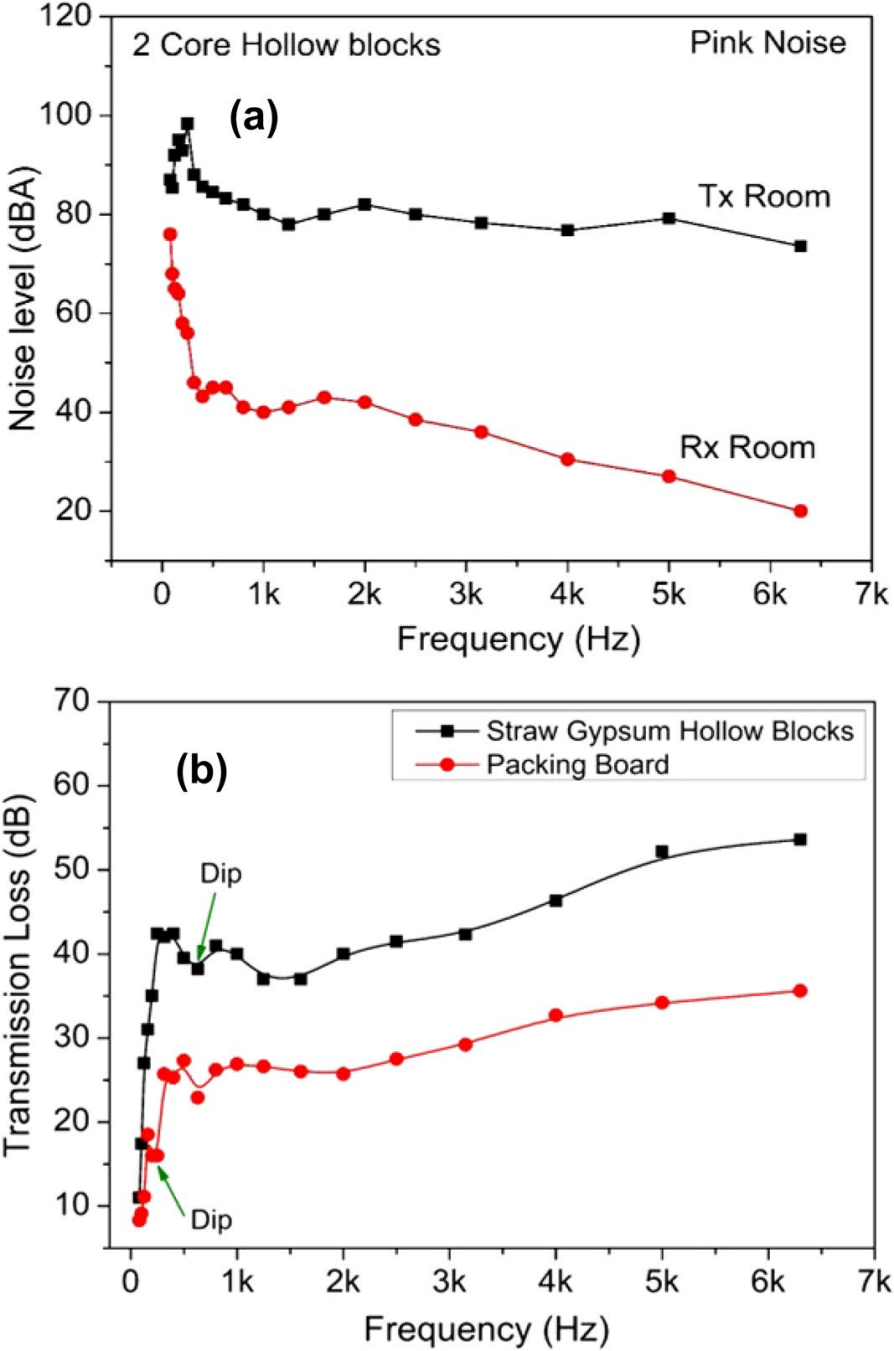
(**a**) Noise level and transmitter and receiver room; and (**b**) STL values of two-core hollow blocks test wall and packing board by reverberation chamber method.

The effect of rice straw addition on STC can be seen in Fig. 8d. It can be observed that the STC value decreases as rice straw content increases. For 5 wt% straw gypsum sample of 20 mm thickness, the STC value is 35 dB and for 25 wt% straw added gypsum sample, the STC value is found to be 28 dB. Construction of testing wall with 10 wt% straw gypsum two-core hollow blocks for STL testing by reverberation chamber method is shown in Fig. 9. STL and corresponding STC values have also been obtained and analysed by reverberation chamber method, as shown in Fig. 10. The individual noise levels in the transmission and receiver room of the reverberation chamber is shown in Fig. 10a. From Fig. 10b, at low frequencies between 80 to 200 Hz, the transmission loss provided by 10 wt% rice straw gypsum two-core hollow blocks lies in the range of 11 to 35 dB. In frequency range of 250 to 500 Hz, the STL values are 40 to 44 dB and follow an increasing trend as the frequency increases. For comparison purpose STL values of sawdust packing board of thickness 10 mm and density 600 kg/m^3^ is also shown in Fig. 10b. From Fig. 10b, the STL values of hollow blocks and sawdust packing boards show a similar trend in STL plots. The initial dip in STL value for straw gypsum hollow block and packing board is around 630 and 200 Hz, respectively. This dip may be attributed to mass-air-mass resonance frequency. An increase in STL values is observed beyond this frequency for both the test walls. Again the second drop in STL values is evident for both wall materials. This second drop in STL values is due to frequency coincidence effect also known as ‘coincidence dip’. This coincidence dip mainly depends upon material’s stiffness and its thickness^40^.

From Fig. 10b, it is evident that the coincidence dip of straw gypsum hollow block occurs at a higher frequency than sawdust packing board. For this reason, the STC value of hollow blocks is higher than packing board. The STC calculation from STL values is done according to ASTM standard^33^ and found to be 40 dB and 21 dB for straw-gypsum hollow block wall and packing board respectively. A similar study on sound reduction index of gypsum hollow panels of 76 mm thickness is done by Junior et al. and have found the sound reduction index value of 32–34 dB which is lower than the two-core hollow straw-gypsum blocks of 200 mm thickness with 70 mm core diameter investigated in the present study^24^.

### Surface spread of flame and fire resistance behaviour

**Figure 11:**
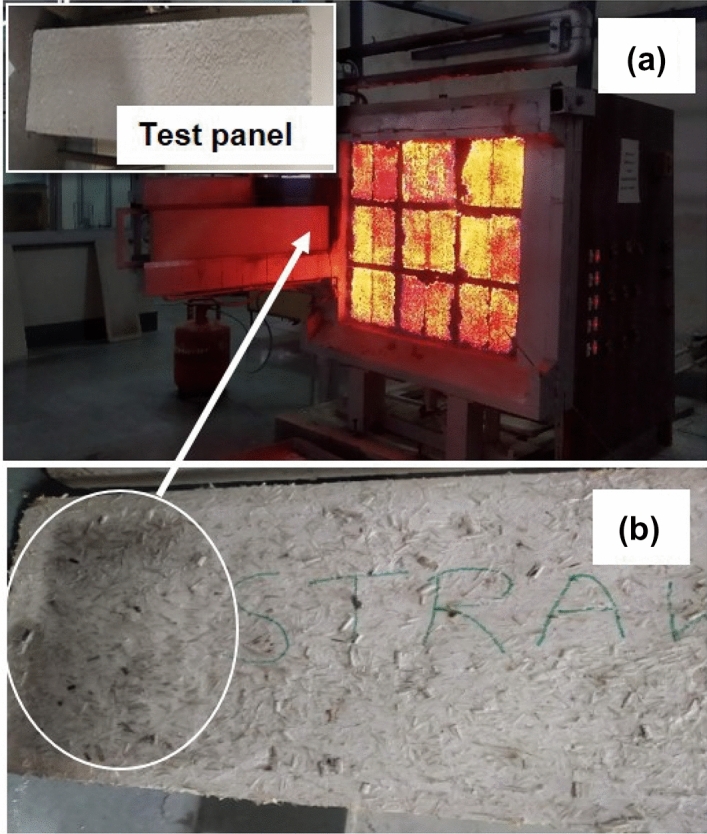
(**a**) Surface spread of flame test on gypsum-agro waste sample, (**b**) surface feature of agro-waste gypsum sample tested for surface spread of flame.

Fire resistance study of agro-waste gypsum samples is of paramount importance which has been investigated in detail in the present study. Rice straw being a combustible organic substance in gypsum blocks/samples may pose a threat to fire hazard and damage the structure. To investigate the fire resistance property, rice straw-based gypsum samples are subjected to fire test according to BS, 476 Part 7, for surface spread of flame^34^ shown in Fig. 11. The experimental run of the surface spread of flame and test panel (in inset) before testing is shown in Fig. 11a. Figure 11b shows no surface spread of flame on the rice straw gypsum board during the test run. Even no chipping or melting is observed during and after the test run and the sample has been classified as class I category. Therefore, as per National Building Code of India (NBCI, Part IV) agro-waste gypsum-based hollow blocks can be used as non-load bearing partition walls^41^.

**Figure 12:**
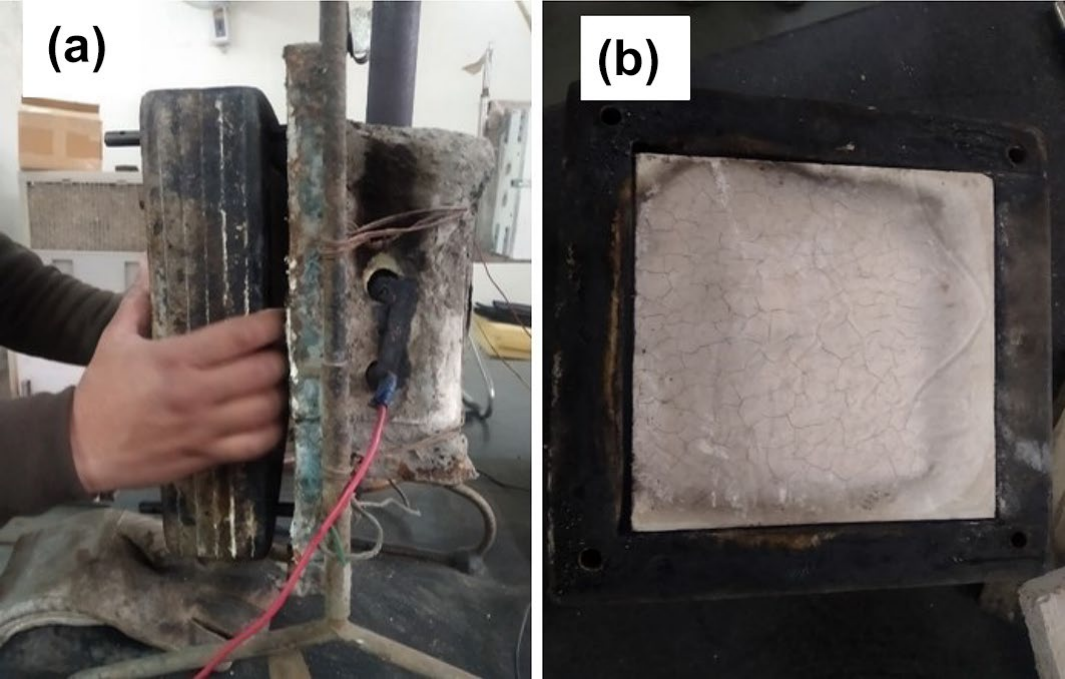
(**a**) Fire propagation index test of agro-waste gypsum samples, (**b**) surface of agro-waste gypsum sample tested for fire propagation index.

Figure 12a shows the agro-waste gypsum-based sample tested for fire propagation test according to British standard^35^. The fire propagation index ($I$) is calculated according to Eq. (4) are found to be 5.078, with sub-indices *i*_*1*_, *i*_*2*_ and *i*_*3*_ are found to be 2.935, 1.73, and 0.413, respectively. According to the BS 476: Part 6 code, if the fire propagation index is less than 12 and sub-index (*i*_*1*_) is less than 6, then the material is classified as fire-resistant. The agro-waste gypsum samples tested here show potential application in fire hazard situation.

From Fig. 12b, it may be observed that the fire propagation index test shows that agro-waste gypsum-based hollow blocks ensure the safety of the material (blocks/building wall) as well as adjacent building components in case of fire with no significant damage observed to the test sample. Few literature have studied the fire resistance properties of gypsum plasterboard with glass fibre content variation. Willams and Mould have shown that for 2 wt% glass fibre gypsum plasterboard, 10% of flexural and 25% of compressive strength was still maintained after exposing the sample at 800 °C for 1-h^42^. Belyachi et al.^43^ studied the surface spread of flame properties on straw fibre composites. They have shown that barley straw pretreated with linseed oil show better fire resistance properties than the water treated straws^43^. In the present study, the untreated rice straws mixed with gypsum also exhibit good fire resistance properties with no surface spread of flame or fire propagation.

### Prototype wall demonstration

**Figure 13:**
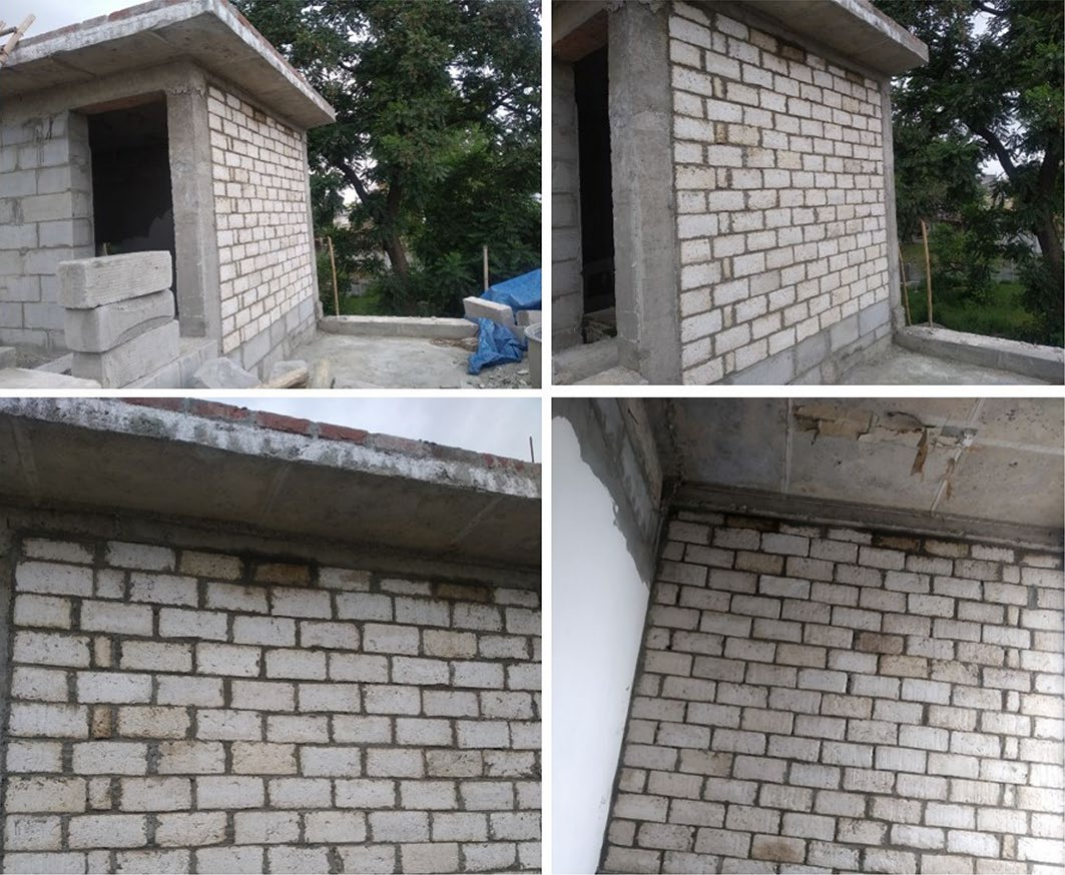
Prototype wall construction from gypsum-agro waste hollow blocks.

A prototype wall of 2.6 m × 2.2 m is constructed in the ground plus one storey building shown in Fig. 13. To check the blocks (wall) performance in adverse conditions, the water and anti- termite repellent coated blocks were kept in 70–90% relative humidity condition for 4 months in an ambient atmosphere. No fungal or termite attack was observed on the surface of blocks. Nail retention test according to IS, 2542 Part 2/Section 8; and thermal transmittance of the prototype wall have also been done^28^. The nail retention test shows 68 N of average force is required as pull-out load. The thermal transmittance (U-value) measurement of AAC block and brick walls was measured by Testo 635 U-value meter. The thermal admittance of 2.5 W/m^2^K is recorded for agro-waste gypsum wall, which is low as compared to brick or concrete wall.

**Figure 14:**
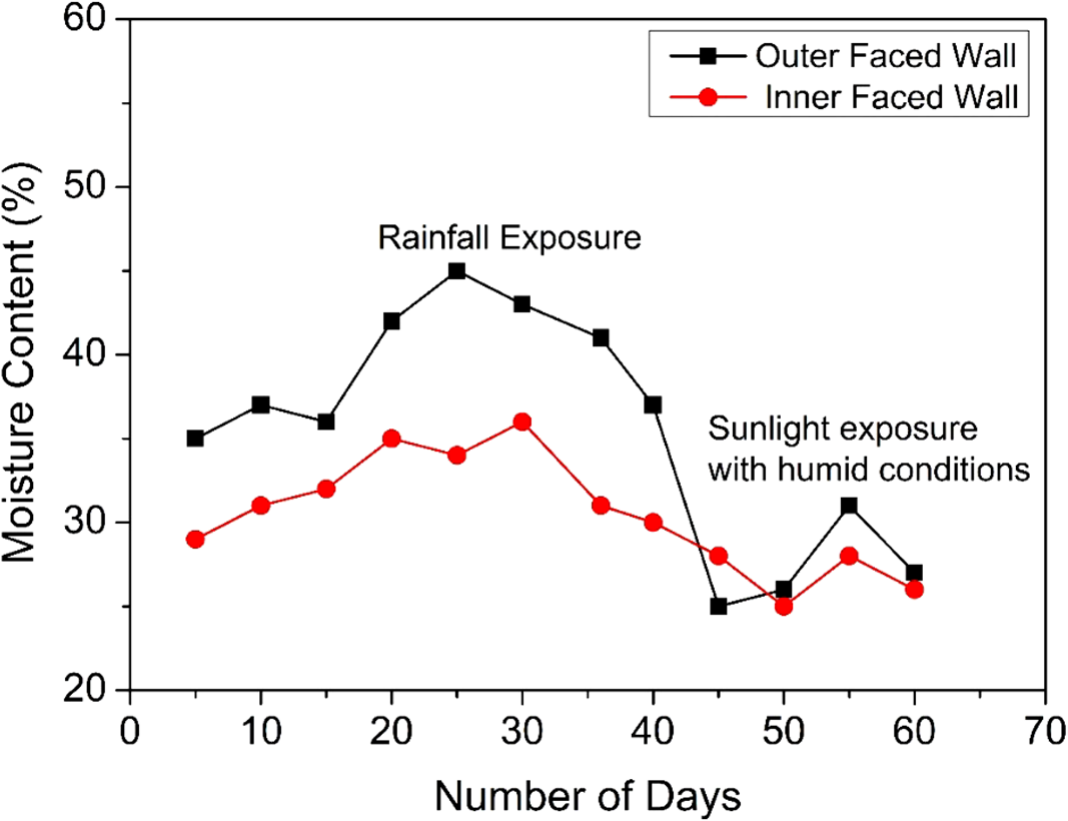
Moisture content variation of agro-waste gypsum wall in rainfall and sunlight conditions with humidity levels of 70–90% in July to August period.

The total amount of agro-waste utilised in this prototype wall construction is approximately 100 kg. With the construction of 10 such similar prototype walls incorporating 1000 kg of agro-waste, around 1000 kg of CO_2_, 60 kg of CO, 200 kg of ash, 2 kg of SO_2_ and nearly 3 kg of particulate matter (PM_2.5_) have been reduced from the environment which otherwise would have caused serious air pollution upon crop residue burning^5^. Figure 14 shows the moisture content in the blocks on both inside and outside wall surfaces from July to August, where relative humidity (Roorkee which falls under composite climate zone) was approximately 70–90%. The wall also faced regular rainfall exposure on the outside surface, causing the lower part of the wall to thoroughly wet. From Fig. 14, it is evident that the maximum moisture content of the outer wall is approximately 45% subjected to intermittent rainfall, whereas minimum of 25% moisture content is observed in sunlight with humid conditions. The inside portion of the wall shows a maximum of 30–35% of moisture content. Various obtained test values and corresponding standards in the present study have been summarised in Table 2.

**Table 2:** Test results of agro-waste gypsum samples and their observed values.

S. No	Parameters	Observed value	Test method
1	Length × height × width Core diameter 70 mm	600 × 300 × 150 mm 300 × 200 × 150 mm	IS 2849: 1983
2	Compressive strength Solid cubes (5 wt%) Hollow blocks (5 wt% straw addition)	9 MPa 2.3 MPa	IS 2542 (Part I) IS 2542 (Part II)
3	Thermal conductivity (5 wt%)	0.16 W/m K	IS: 3346–1980
4	Noise reduction coefficient (NRC)	24%	ASTM E 1050
5	Sound absorption average (SAA)	26%	ASTM E 1050
6	Sound transmission loss/class (impedance tube) 20 mm thick	34 dB	ASTM E 2611
7	Sound transmission class (reverberation chamber method)	40 dB	ASTM E 413
8	Evaluation for fire propagation	Class I	BS 476 Part 6
9	Classification of the surface spread of flame	Class I	BS 476 Part 7
10	Water absorption (24 h)/after treatment	22% / 15–18%	IS 2542 (Part II)

## Limitations, policy implications and future prospects

As far as the current study is concerned, there are many opportunities and challenges for stubble management. The first and foremost challenge in crop residue management is the lack of awareness among the farmers towards environmental and health aspects of current and future generations. According to a study, 60% rise in fire counts due to stubble burning has been recorded since the breakout of COVID-19 pandemic^44^. The rise in stubble burning cases may be attributed to lack of manpower on-farm sites and farmers' low income. This is a very serious concern that needs to be addressed through proper education and awareness. According to Government of India a ‘National Policy for Management of Crop Residue (NPMCR)’ has been made with objectives viz., control of stubble burning to prevent environmental pollution and minimizing soil nutrients loss, utilisation of crop residue for various purposes like charcoal gasification, power generation, production of bio-ethanol and as packing material for paper, board and panel industries^45^.

Regarding policy implications for managing the crop residue, the farmers should be encouraged to adopt clean agriculture practices and holistic approaches which can provide eco-friendly solutions to these problems. One such approach has been demonstrated in the present study. By managing crop residues, the current approach will help farmers to have an additional income. It will definitely help small-scale block making industries to grow and manufacture eco-friendly and sustainable products.

## Conclusions

The properties of agro-waste-based gypsum samples with various straw sizes, temperature and agro-waste content variation of 0 to 25 wt% were investigated. The present study has shown the potential utilisation of agro-waste (rice straw) in gypsum hollow-core blocks for partition walls. Various composition of agro-waste-based gypsum samples have been tested and studied for mechanical, thermal, sound absorption, sound transmission loss, fire retardant, nail retention, thermal admittance studies by Indian, ASTM and British standards. The performance evaluation of the prototype wall has been done under adverse environments of high humidity variations, rainfall, and sunlight exposure. The following conclusions can be made from the current study.

As the agro-waste content increases from 0 to 25 wt%, the density of gypsum samples decreases from 1270 to 830 kg/m^3^, making it lightweight material that can be utilised in non-load bearing wall application. Compressive strength varies over 10 to 2 MPa and 3.8 to 0.5 MPa for solid gypsum cubes, and two-core straw added hollow gypsum blocks for 0 to 25 wt% rice straw content addition. The effect of reduced straw size shows enhancement in the compressive strength due to uniform mixing and strong bonding in the gypsum matrix. The thermal conductivity of straw gypsum hollow core block shows a decreasing trend with the increase in rice straw content from 0.2 to 0.11 W/m K. The wall constructed from these blocks can provide good thermal comfort in hot weather conditions. One of the drawbacks of the gypsum material is its high water absorption capacity which can be further aggravated by rice straw incorporation due to the porous nature of straw. Through external treatment viz., water repellent coating, the 24-h water absorption value was brought down over the range of 15 to 18% range from 22%.

Acoustic study on agro-waste gypsum samples reveals that NRC increases from ~ 25 to 45% with rice straw addition from 0 to 25 wt%. Moreover, the STC values decrease from 37 to 28 dB as the agro-waste content increases. This reduction in STC value is attributed to increased porosity due to rice straw incorporation. The STC rating from the reverberation chamber study of hollow-core gypsum blocks is ~ 40 dB which is higher than sawdust packing board material.

The fire-resistant properties viz., surface spread of flame, and fire propagation investigation have shown promising results for the rice straw-based gypsum sample. The 5 wt% straw-gypsum sample qualifies for the class I category with no surface spread of flame, chipping or melting is observed. The fire propagation index is found to be 5.078, with sub-indices *i*_*1*_, *i*_*2,*_ and *i*_*3*_ are found to be 2.935, 1.73 and 0.413, respectively. With fire propagation index (*I*) less than 12, and sub-index (*i*_*1*_) less than 6, indicates that the agro-waste gypsum material may be classified as potential fire-resistant material.

The prototype wall constructed from hollow blocks was exposed to high relative humidity of 70–90% and precipitation conditions after water repellent and anti-termite coating. The wall has shown negligible leaching, termite, or fungal attack degradation. The nail retention test shows 68 N of average force is required as the pull-out load from the prototype wall. The thermal admittance of 2.5 W/m^2^K is recorded for agro-waste gypsum wall which is low as compared to brick or concrete wall. The whole idea behind this study was to manage the issue of crop residue burning, which has increased during COVID-19 pandemic. The total amount of agro-waste utilised in the construction of the prototype wall is approximately 100 kg. Utilisation of ~ 1000 kg of agro-waste in gypsum blocks would require 10 such similar prototype walls would have reduced approximately 1000 kg of CO_2_, 60 kg of CO, 200 kg of ash, 2 kg of SO_2_ and nearly 3 kg of particulate matter (PM_2.5_) emission by 1000 kg of crop residue burning^5^. The agro-waste based gypsum blocks may be used a promising material for drywall partitions owing to their thermal insulation, moderate density, good acoustic and fire-resistant properties. The of crop residue burning is causing serious health issues which is very detrimental in COVID-19 pandemic situation. Clean and eco-friendly agricultural practices and awareness among farmers about ill-effects of crop residue burning and implementation of government policies are required to mitigate the effects of crop residue burning for a clean and sustainable future.

## Abbreviations

IS: Indian standard
RH: Relative humidity
ASTM: American society for testing and materials
NRC: Noise reduction coefficient
SAA: Sound absorption average
STL: Sound transmission loss
STC: Sound transmission class
SLM: Sound level meter
BS: British standard
NBCI: National building code of India
PM: Particulate matter
